# Hypoxia Inhibits Osteogenesis and Promotes Adipogenesis of Fibroblast-like Synoviocytes via Upregulation of Leptin in Patients with Rheumatoid Arthritis

**DOI:** 10.1155/2022/1431399

**Published:** 2022-12-07

**Authors:** Menglei Ding, Yu Cheng, Zhen Xu, Ying Lu, Jing Li, Liu Lu, Ming Zong, Lieying Fan

**Affiliations:** Department of Clinical Laboratory, Shanghai East Hospital, Shanghai Tongji University School of Medicine, Shanghai 200120, China

## Abstract

Hypoxia is associated with the pathogenesis of rheumatoid arthritis (RA). RA fibroblast-like synoviocytes (FLSs) are able to differentiate into osteoblasts and adipocytes. In this study, we aimed to investigate the role of hypoxia in the osteogenesis or adipogenesis of RA-FLSs. Bioinformatics analysis was performed to profile gene expression in the datasets of GSE21959, GSE32006, and GSE55875, and flow cytometry was performed for FLS characterization, while Alizarin Redand Oil Red O staining for osteogenic or adipogenic differentiation of FLSs, respectively. RNA interference leptin knockdown was used to determine the role of leptin in the osteogenesis and adipogenesis of RA-FLSs, and the expression of osteogenic and adipogenic markers was quantified by RT-qPCR and Western blotting. FLSs exhibited a mesenchymal stem cell (MSC)-like phenotype and we observed a limited self-renewal capacity in RA-FLSs compared to that in MSCs, but it was still greater than osteoarthritis (OA)-FLSs. Hypoxia did not change the RA-FLS MSC-like phenotype but inhibited the osteogenic differentiation and promoted the adipogenic differentiation of RA-FLSs. From the bioinformatics analysis ofGSE21959, GSE32006, and GSE55875 datasets, we found leptin, the only perturbed hypoxia-mediated upregulated gene across the three profiled datasets. Leptin knockdown in RA-FLSs reversed the hypoxia-mediated reduction of osteogenesis and hypoxia-mediated enhancement of adipogenesis by elevated expression of osteogenic markers and reduced expression of adipogenic markers, respectively. Therefore, hypoxia-leptin regulation of the osteogenic and adipogenic differentiation of RA-FLSs advances our understanding of RA pathogenesis, meanwhile also provides opportunities for future therapeutic intervention of RA.

## 1. Introduction

Rheumatoid arthritis (RA) is a systemic autoimmune disease that affects approximately 0.28% population of China [1]. RA is characterized by articular erosion and defective repair, leading to functional disability [2]. Most of the current therapies for RA focus on the attenuation of osteoclast-mediated bone resorption, but not the increase of bone formation by osteoblasts. Therefore, many RA patients have inadequate bone mass.

It was reported that RA patients had low oxygen concentration in the disease joint cavity compared to the normal joint cavity, and hypoxia was inversely correlated with the joint inflammation responsible for disease activity [3]. However, the effects of hypoxia on osteogenesis and adipogenesis in RA joints remain controversial [4]. There were studies demonstrating that reduced oxygen tension promoted human bone marrow-derived mesenchymal stem cells (BM-MSCs) differentiation into osteoblasts, which had osteogenic markers such as runt-related transcription factor 2 (RUNX2), osteocalcin (OCN), and alkaline phosphatase (ALP) [5, 6], while hypoxia was reported to inhibit the osteogenesis of BM-MSCs [7]. There were conflicting results of hypoxia for adipogenic differentiation of BM-MSCs with either enhancement or suppression [5, 8–10].

RA fibroblast-like synoviocytes (RA-FLSs) are located in the intimal lining of the joint, which are the principal sources of proinflammatory cytokines such as IL-6 and TNF-*α*leading to joint inflammation that has been implicated in the joint destruction of RA [11, 12]. RA-FLSs are originated from BM-MSCs but possess distinct characteristics [13, 14]. RA-FLSs could secrete abundant receptor activator of nuclear factor-*κ*B ligand (RANKL) andpromote osteoclast maturation for bone resorption [11, 15]. Persistent hypoxic milieu is responsible for the transformation of RA-FLSs. Our previous studies demonstrated that hypoxia caused increased proliferation but reduced apoptosis of RA-FLSs [16, 17]. However, the exact roles of RA-FLSs in osteogenesis for bone repair remain elusive.

Here, we characterized the RA-FLSs under normoxia and hypoxia conditions and found that hypoxia attenuated osteogenesis but promoted adipogenesis of RA-FLSs. We performed bioinformatics analysis and identified leptin as the target gene for the hypoxia-induced imbalance of RA-FLS osteoblast-adipocyte differentiation. Further, leptin inhibition by siRNA knockdown reversed the reduced RA-FLS osteoblast differentiation, and reversed the increased RA-FLS adipocyte differentiation to decrease adipose formation. Forced RA-FLSs differentiation into osteoblasts to repair the bone defect in situ could be a future therapeutic option for RA.

## 2. Materials and Methods

### 2.1. Isolation and Culture of Primary FLSs from RA and Control Patients

Primary FLSs were obtained from the synovial tissues of three RA patients with FLSs from three OA patients who underwent knee arthroscopy or replacement surgery at Shanghai East Hospital as the control FLSs. Freshly obtained synovial tissues from RA or OA patients needed to be processed within 4 h. The tissues were rinsed, minced, and evenly spread on the bottom of cell culture flasks in RPMI 1640 medium (Life Technologies, Carlsbad, CA, USA) at 37°C for 6 h. Next, the tissues were cultured in DMEM medium supplemented with 10% fetal bovine serum (FBS) and penicillin (100 U/ml) and streptomycin (100 *μ*g/ml), and incubated at 37°C in a humidified atmosphere of 5% CO_2_. Nonadherent tissue pieces were removed and adherent cells were replenished with the fresh medium every 3 to 5 days. The primary FLSs were passaged when reaching 70–80% confluence. The FLSs were cultured under normoxia (21% O_2_) and/or hypoxia (3% O_2_) at 37°C conditions in the following experiments. The written informed consent was obtained from each participant before the collection of synovium samples following the protocol approved by the ethics committee of Shanghai East Hospital.

### 2.2. Characterization of FLSsby Flow Cytometry

Antibodies against human antigens, CD90-APC, CD73-PE-Cy5.5, CD105-BV421, or CD45-FITC were purchased from Becton Dickinson Biosciences (BD Biosciences, San Jose, CA, USA). Resuspended cells were incubated with antibodies for 30 minutes at room temperature in the dark followed by flow cytometry on FACS Aria II flow cytometer (BD, Franklin Lakes, NJ, USA) and then analyzed with FlowJo software (Tree Star, Ashland, OR, USA).

### 2.3. Osteogenic and Adipogenic Differentiation of FLSs

Mesenchymal stem cell osteogenesis kit (Cat. No. SCR028; Millipore, Temecula, CA, USA) was used to induce the osteogenic differentiation of FLSs following the manufacturer's instructions. Alizarin Red was used to stain calcium deposits of the osteoblasts. Adipocytes generated from primary FLSs were induced by mesenchymal stem cell adipogenesis kit (Cat. No. SCR020; Millipore, Temecula, CA, USA) following the manufacturer's instructions. Oil Red O solution was then added to stain the lipid droplets of adipocytes. For 3-day induction, the osteogenic induction medium (OIM) contained 10% FBS, 0.5 *μ*M dexamethasone, 1 mM ascorbic acid 2-phosphate solution, 50 mM glycerol 2-phosphate solution, 1× L-glutamine, 1× penicillin-streptomycin, the adipogenic induction medium (AIM) contained 10% FBS, 5 *μ*M dexamethasone, 2.5 mM 3-isobutyl-1-methylxanthine, 50 *μ*g/ml insulin, 1 mM indomethacin. 1× penicillin-streptomycin.

### 2.4. RNA Extraction and RT-qPCR

We quantified mRNA levels of RUNX2, OCN, ALP, peroxisome proliferator activated receptor *γ* (PPAR*γ*), CCAAT enhancer-binding protein *α* (C/EBP*α*), and fatty acid binding protein 4 (FABP4) by RT-qPCR.

Briefly, total RNA was extracted using an RNAiso plus kit (Takara, Tokyo, Japan). Complementary DNA (cDNA) was generated from 1 *μ*g total RNA using a Prime Script RT Reagent Kit (Promega, Corporation, Wisconsin, USA). A SYBR Premix Ex Taq II kit (Takara, Liaoning, China) was used to quantify mRNA levels of interest genes in the ABI7500 Real-Time PCR System. The qPCR running conditions were as follows: predenaturation at 95°C for 10 m, 40 cycles of 95°C for 10 s, and 60°C for 60 s. The gene expression was normalized to *β*-actin expression. The relative expression of each gene was normalized to control by standard 2-^∆∆^CT calculation. The primers (Table 1) were designed and synthesized by Sangon (Shanghai Sangon Biological Engineering Technology & Services Company, China).

### 2.5. Colony Formation Assay

The FLSs were seeded into 24-well plates at a density of 5 × 10^2^ cells per well and cultured in complete low-glucose DMEM medium for two weeks. The medium was replaced every five days until two weeks. Cells were then fixed in 4% paraformaldehyde and rinsed with PBS. Thereafter, 0.1% (w/v) crystal violet was added to the cells and then removed after 15 minutes. The minicolonies with 20–30 cells were counted using an inverted microscope (Leica).

### 2.6. Datamining Gene Expression Profile from Datasets of GSE21959, GSE32006, and GSE55875

Gene expression profile datasets of GSE21959, GSE32006, and GSE55875 were downloaded from the Gene Expression Omnibus (GEO) database (http://www.ncbi.nlm.nih.gov/geo/). We selected 9 mRNA samples of RA-FLSs incubated for 22 hours under normoxic or hypoxic (0.5% O_2_) conditions from GSE21959. On the other hand, both GSE32006 and GSE55875 microarray datasets contained 3 mRNA samples of BM-MSCs exposed to 0.5% or 21% O_2_ for 24 hours.

The gene expression in the three datasets was normalized using Sangerbox tool (http://www.sangerbox.com), a free online platform for data analysis. We then used the limma package of the Sangerbox tool to evaluate the differentially expressed genes (DEGs) in each dataset. The DEGs were selected using an adjusted *p* value of <0.05 and ∣log2 (fold change)  | ≥2. To show overlapping DEGs, if any, a Venn diagram was drawn using a web-based online tool (http://bioinformatics.psb.ugent.be/webtools/Venn). The procedure was shown in supplementary figure 1.

### 2.7. RNA Interference and Transfection

Three small interfering RNAs (siRNAs) against human leptin (siLep1: 5′-UCCUGACCUUAUCCAAGAUTT-3′; siLep2: 5′ -GGAACUCCCAGCAACACAATT-3′); si-Lep3: 5′ -GGAGAGUACAGUGAGCCAATT-3′) and negative control scramble siRNA (Con: 5′-TTCTCCGAACGTGTCACGT-3′) were purchased from Genepharma (Shanghai, China). siRNA transfection was performed using Lipofectamine 3000 (Invitrogen, Carlsbad, CA, USA) according to the manufacturer's instructions.

### 2.8. Western Blot Analysis

Western blotting was performed as described previously [16]. Primary antibodies were purchased from Santa Cruz (Leptin: sc-484C8; *β*-Actin: sc-47778; and OCN: sc-74495) and cell signaling technology (RUNX2: 12556; C/EBP*α*: 8178; and PPAR*γ*: 2435). The blots were visualized using BeyoECL Star (BEYOTIME, China) and the bands were visualized with a Gel-imaging workstation (Tiangen, Shanghai, China).

### 2.9. Immunohistochemistry Analysis

Paraffin-embedded sections of synovium tissues from the patients of RA or OA were prepared and subjected to immunohistochemical analysis. We incubated the sections with leptin primary antibody and then secondary antibody (Envision™Detection Kit, Dako). We visualized the bands using a diaminobenzidine substrate kit (Dako) following the manufacturer's instructions.

### 2.10. Statistical Analysis

Statistical analysis was performed using GraphPad Prism 7 (GraphPad Software, La Jolla, CA, USA). All experiments were repeated three times. Data were presented as mean ± standard error (SEM). We used unpaired Student's *t*-test to compare two different groups. If the variance unequal, we made a Welch's correction. A *p* value less than 0.05 were considered as statistically significant.

## 3. Results

### 3.1. The Characteristics of FLSs with Mesenchymal Stem Cell (MSC)-like Phenotype and RA-FLSs Having a Limited Self-Renewal Capacity but Greater Osteogenic and Adipogenic Capacity

**Figure 1:**
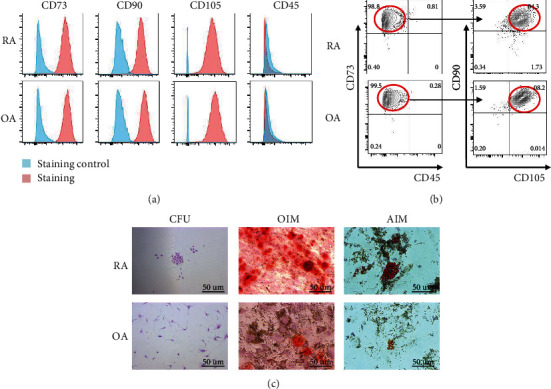
RA-FLSs show a MSC-like phenotype and are able to differentiate into osteoblasts or adipocytes. (a) Surface antigen profiles of RA-FLSs and OA-FLSs. The red line represents the specific antibody while the blue line represents the isotype control. (b) Analysis of the surface antigens with FlowJo software. (c) Crystal violet staining of RA-FLSs and OA-FLSs after culture for 14 days under normoxic condition. Alizarin Red staining of RA-FLSs and OA-FLSs after culture in OIM for 21 days under normoxic condition. Oil Red O staining of RA-FLSs and OA-FLSs after culture in AIM for 21 days under normoxic condition. All experiments were repeated three times. CFU: cell formation unit; OIM: osteogenic induction medium; AIM: adipogenic induction medium. Scale bars: C = 50 *μ*m.

RA-FLSs and OA-FLSs isolated from the synovium of RA and OA patients, respectively, were morphologically heterogeneous. In order to confirm the MSC-like characteristics of FLSs, we analyzed the expression of mesenchymal lineage-associated cell surface markers. Both RA-FLSs and OA-FLSs were positive for CD73, CD90, and CD105 and negative for CD45 (Figure 1(a)). The proportion of CD73^+^CD90^+^CD105^+^CD45^−^ RA-FLSs or OA-FLSs was more than 90% (Figure 1(b)). In addition, the colony formation assay showed RA-FLSs yielded fewer colonies than MSCs [18], but more colonies than OA-FLSs (Figure 1(c)), which indicated a limited self-renewal capacity in RA-FLSs compared to that in MSCs, but it was still greater than OA-FLSs. Moreover, we assessed the osteogenic and adipogenic differentiation of FLSs. After 21 days of induction, the RA-FLSs exhibited a greater capacity to differentiate into osteoblasts or adipocytes compared to OA-FLSs (Figure 1(c)). These data suggest that RA-FLSs have a greater capacity of osteoblast or adipocyte differentiation compared to OA-FLSs.

### 3.2. Hypoxia Maintains the Self-Renewal Capacity, Inhibits Osteogenic Differentiation but Promotes the Adipogenic Differentiation of RA-FLSs

**Figure 2:**
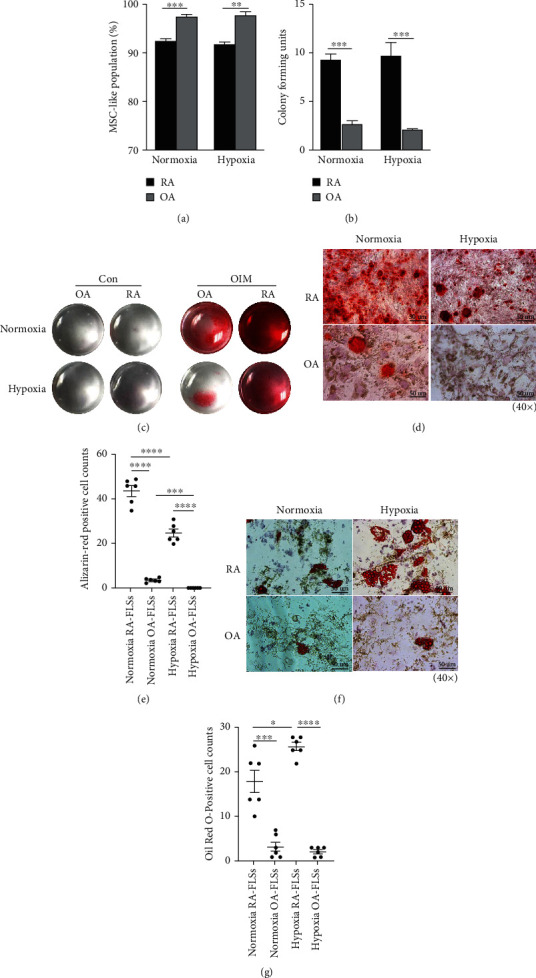
Maintenance of the phenotype and self-renewal capacity of RA-FLSs under hypoxic condition and Hypoxia inhibits osteogenesis but increases adipogenesis of RA-FLSs. (a) Percentage of MSC-like cells of the RA-FLSs and OA-FLSs exposed to normoxic and hypoxic conditions for 48 hours (three independent experiments). (b) Colony formation assays of RA-FLSs and OA-FLSs exposed to normoxic and hypoxic conditions for 14 days. Data quantification is mean ± SEM. (*t*-test, three independent experiments). (c, d) RA-FLSs and OA-FLSs induction of osteoblast differentiation after culture in OIM for 21 days, and then stained by Alizarin Red solution. (e) Alizarin Red positive cells per field were counted. Data quantification is mean ± SEM. (*t*-test, six randomized fields, three independent experiments, and one representative experiment shown). (f) RA-FLSs and OA-FLSs induction of adipocyte differentiation after culture in AIM for 21days, and then stained with Oil Red O solution. (g) Oil O Red positive cells per field were counted. Data quantification is mean ± SEM. (*t*-test, six randomized fields, three independent experiments, and one representative experiment shown). ^∗^*p* < 0.05, ^∗∗∗^*p* < 0.001, ^∗∗∗∗^*p* < 0.0001. Scale bars: C = 50 *μ*m.

Hypoxia is a typical feature of the diseased synovium in RA patients. Therefore, we first examined the effects of hypoxia on the colony formation capacity of RA-FLSs or OA-FLSs. Flow cytometry results showed thatRA-FLSs under hypoxic condition exhibited more MSC-like cells (CD73^+^CD105^+^CD90^+^CD45^−^ population) (Figure 2(a)) and had more colony formation than OA-FLSs (Figure 2(b)). These results demonstrated that RA-FLSs retain self-renewal capacity under hypoxia conditions. Next, we investigated the impact of hypoxia on the osteogenic and adipogenic differentiation capacity of FLSs. Alizarin Red was used to stain the calcium deposits of cells. The results showed an absence of staining in control groups cultured with regular medium under normoxia and hypoxia conditions, which indicated no spontaneous osteogenic differentiation of FLSs (Figure 2(c)). A dramatic decrease of the staining was observed in both RA-FLSs and OA-FLSs under hypoxic conditions (Figures 2(c) and 2(d)), which indicated that hypoxia reduced osteogenic capacity. However, we found that RA-FLSs under hypoxic conditions exhibited greater osteogenic capacity than OA-FLSs (Figures 2(c)–2(e)).

In addition, adipogenesis of FLSs was evaluated by Oil Red O staining. The results showed that RA-FLSs exhibited more lipid accumulation with larger lipid droplets after 21 days of culture under hypoxic conditions, while hypoxia exerted little impact on the adipogenesis of OA-FLSs since they barely differentiated into adipocytes under both hypoxic and normoxic conditions (Figures 2(f) and 2(g)).

These studies demonstrate that hypoxia inhibits the osteogenic differentiation of RA-FLSs and OA-FLSs, but promotes the adipogenic differentiation of RA-FLSs.

### 3.3. Leptin Is Upregulated in RA-FLSs under Hypoxic Condition

**Figure 3:**
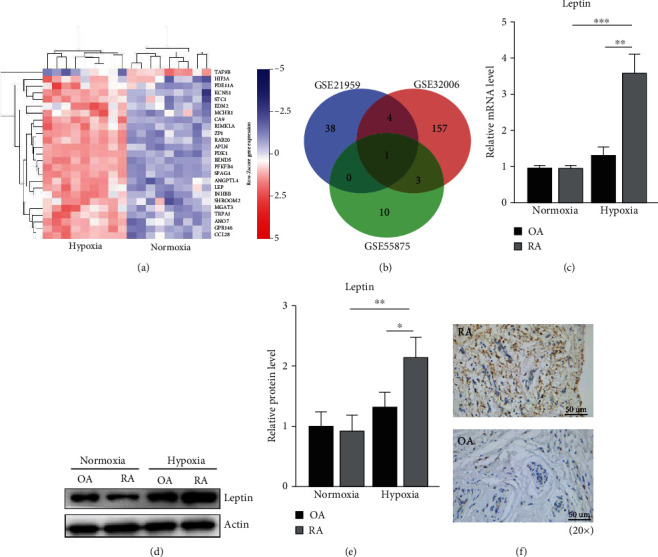
Hypoxia increases leptin expression of RA-FLSs. (a) Heat map shows the hierarchical clustering of RA-FLSs cultured under hypoxic and normoxic conditions. (b) Venn diagram illustrates the overlap of DEGs data from three independent analyses. (c) RT-qPCR analysis shows the expression of LEPin RA-FLSs and OA-FLSs after 48 hr hypoxia or normoxia treatment. The experiments were repeated three times. Data quantification is mean ± SEM. (d, e) Western blot shows leptin expression in RA-FLSs and OA-FLSs after 48 hr hypoxia or normoxia treatment. *β*-Actin was used as an internal control. The experiments were repeated three times. Data quantification is mean ± SEM. (f) The distribution of leptin in synovium samples obtained from patients with RA or OA. ^∗^*p* < 0.05, ^∗∗^*p* < 0.01, ^∗∗∗^*p* < 0.001. Scale bars: C = 50 *μ*m.

There are studies that reported a positive correlation between insufficient osteogenesis with bone damage in RA [19, 20]. In addition, Li and Mckarov demonstrated that RA-FLSs contained a substantial fraction of BM-MSCs [14]. Moreover, we found RA-FLSs in patients showed MSC-like phenotype (Figure 1). Therefore, GSE21959, GSE32006, and GSE55875 microarray datasets from GEO were analyzed for altered genes under hypoxic condition compared to normoxic condition. The FLSs in GSE21959 datasets were derived from RA synovium samples, while GSE32006 and GSE55875 datasets studied BM-MSCs from the patients. A total of twenty-nine genes were upregulated while one gene was downregulated in GSE21959 (Figure 3(a)). Interestingly, leptin (LEP) was the only upregulated gene at the intersection of DEGs in the three datasets (Figure 3(b)). We confirmed the upregulation of the leptin mRNA and protein in RA-FLSs under hypoxicconditions by RT-qPCR and Western blot, while no significant change was observed in OA-FLSs (Figures 3(c)–3(e)). Moreover, immunohistochemical analysis indicated that leptin predominantly localized in the cytoplasm of synovium samples obtained from the RA or OA patients, and the expression significantly increased in RA patients (Figure 3(f)). These results suggest that leptin is upregulated in RA-FLSs under hypoxic conditions, which indicates that leptin might be important in the regulation of hypoxia-mediated FLS differentiation possibly associated with RA pathogenesis.

### 3.4. Leptin Knockdown Reverses the Hypoxia-Mediated Reduction of Osteogenesis of RA-FLSs

**Figure 4:**
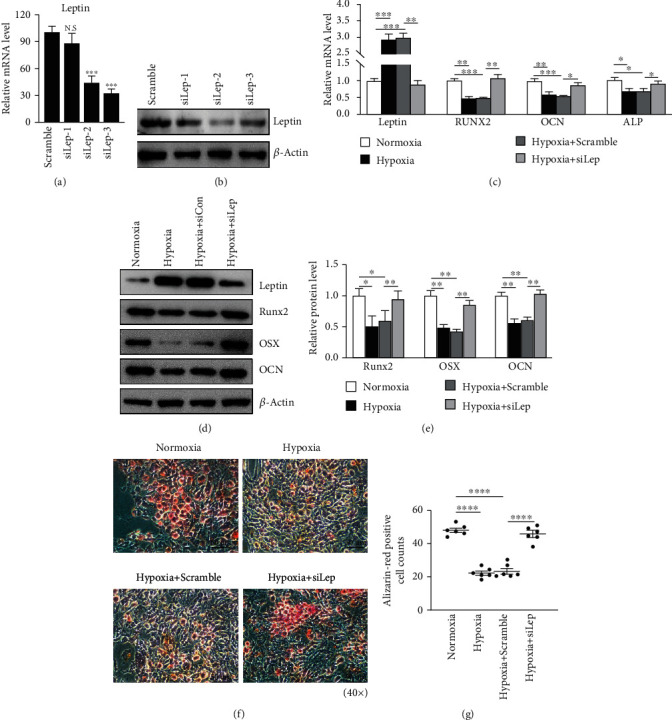
Knockdown of leptin reversed the hypoxia-mediated reduction of osteogenesis of RA-FLSs under hypoxia condition. (a) The knockdown efficacy of siRNAs targeting leptin evaluated by RT-qPCR. NC: Negative Control; siLep: siRNA-leptin. Data quantification is mean ± SEM (*t*-test, three independent experiments). (b) The absence of leptin in RA-FLSs was analyzed by Western blot. Data quantification is mean ± SEM (*t*-test, three independent experiments). (c) RT-qPCR analysis of *leptin* and osteogenesis-related genes (Runx2, Ocn, and Alp) in leptin-knockdown or scrambled siRNA control RA-FLSs after culture with osteogenic induction medium for 3 days under hypoxia and normoxia condition. Data quantification is mean ± SEM (*t*-test, three independent experiments). (d, e) Westernblot shows leptin expression and osteogenesis-related proteins (RUNX2, OSX, and OCN) in leptin-knockdown or scramble siRNA control RA-FLSs after culture with osteogenic induction medium for 3 days under hypoxia and normoxia condition. The expression normalized with *β*-actin. Data quantification is mean ± SEM (*t*-test, three independent experiments). (f) Alizarin Red staining of the leptin-knockdown or scrambled siRNA control RA-FLSs after culture with osteogenic induction for 21 days under hypoxia and normoxia conditions. (g) Alizarin Red positive cells per field were counted. Data quantification is mean ± SEM. (*t*-test, six randomized fields, three independent experiments, and one representative experiment shown). ^∗^*p* < 0.05; ^∗∗^*p* < 0.01; ^∗∗∗^*p* < 0.001; ^∗∗∗∗^*p* < 0.0001. Scale bars: C = 50 *μ*m.

To further study the impact of leptin on the changes of FLSs under hypoxic conditions, three siRNAs targeting leptin (siLep-1, siLep-2, and siLep-3) were used to silence leptinexpression in RA-FLSs and siLep-2 was observed having the highest inhibitory efficacy (Figures 4(a) and 4(b)).

Leptin siLep-2-knockdown RA-FLSs and control scramble siRNA RA-FLSs were cultured in regular culture medium or OIM under hypoxic conditions. RT-qPCR results showed a significant upregulation of leptin mRNA and protein expression of RA-FLSs under hypoxic conditions, while osteoblast markers (RUNX2, OCN, and ALP) were significantly downregulated (Figures 4(c)–4(e)), which indicated that hypoxia increased leptin expression and reduced RA-FLS osteogenesis. Suppression of leptin significantly enhanced the expression ofosteoblast markersin mRNA and protein levels (Figures 4(c)–4(e)). Moreover, a significant increase in calcium deposits and mineralization was observed in the leptin-knockdown RA-FLSs in comparison with the control group (Figures 4(f) and 4(g)). These results suggest that leptin is important in the regulation of hypoxia-mediated reduction ofRA-FLS osteogenesis.

### 3.5. Leptin Knockdown Attenuates the Hypoxia-Mediated Elevated Adipogenesis of RA-FLSs

**Figure 5:**
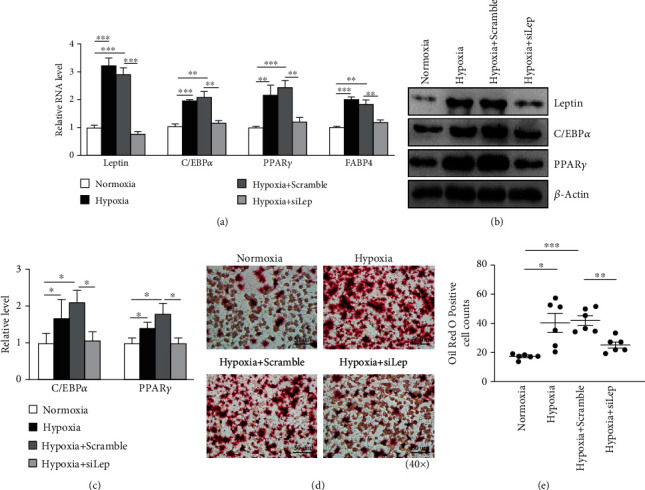
Knockdown of leptin attenuated the hypoxia-mediated increase of adipogenesis of RA-FLSs under hypoxia conditions. (a) RT-qPCR analysis of *leptin* and adipogenesis-related genes (C/EBP*α*, PPAR*γ*, and FABP4) in leptin-knockdown or scrambled siRNA control RA-FLSs after culture with adipogenic induction medium for 3 days under hypoxia and normoxia conditions. Data quantification is mean ± SEM (*t*-test, three independent experiments). (b, c) Westen blot analysis of leptin and adipogenesis-related proteins (C/EBP*α* and PPAR*γ*) in leptin-knockdown or scrambled siRNA control RA-FLSs after culture with adipogenic induction medium for 3 days under hypoxia and normoxia conditions. The expression normalized with *β*-actin (*t*-test, three independent experiments). (d) Oil Red O staining of leptin-knockdown or scramble siRNA control RA-FLSs after culture with adipogenic induction medium for 21 days under hypoxia and normoxia conditions. (e) Oil Red O positive cells per field were counted. Data quantification is mean ± SEM. (*t*-test, six randomized fields, three independent experiments, and one representative experiment shown). ^∗^*p* < 0.05; ^∗∗^*p* < 0.01; ^∗∗∗^*p* < 0.001. Scale bars: C = 50 *μ*m.

In consistent with the upregulation of leptinexpression in RA-FLSsafter 3-day adipogenic induction and under hypoxic conditions, the expression of adipogenic markers, C/EBP*α*, PPAR*γ*, and FABP4 was also elevated, which indicated the increase of RA-FLS adipogenesis (Figures 5(a)–5(c)). Leptin knockdown significantly suppressed the expression of C/EBP*α*, PPAR*γ*, and FABP4 in RA-FLSs (Figures 5(a)–5(c)). Moreover, we found that leptin knockdown significantly inhibited the formation of hypoxia-inducible lipid droplets of RA-FLSs (Figures 5(d) and 5(e)). These results suggest that leptin is important in the regulation of hypoxia-mediated enhancementof RA-FLS adipogenesis.

## 4. Discussion

Hypoxia is associated with RA pathogenesis; however, its role in RA-FLS differentiation remains undefined. Our previous studies revealed that hypoxia stimulated proliferation and autophagy in RA-FLSs [16, 17]. Hypoxia is a distinctive feature of the RA milieu, as well as the BM-MSC microenvironment. A recent review showed that hypoxia has double-edged effects on the fate of BM-MSCs and both positive and negative associations with osteogenesis and adipogenesis have been observed [4]. In this study, we explored the impact of hypoxia onosteoblast and adipocyte differentiation capacity of RA-FLSs. The results showed that RA-FLSs possess a MSC-like phenotype, and hypoxia had no influence on the phenotypic changes, which is in consistent with the previous studies [21, 22]. Moreover, Oil Red O and Alizarin Red staining indicated that RA-FLSs during hypoxia exhibited reduced osteoblast differentiation resulting in a lower rate of bone formation, and increased adipocyte differentiation resulting in more adipose formation in comparison to OA-MSCs. These findings revealed an association of RA-FLSs with the balance of bone and adipose formation during hypoxia, demonstrating a novel effect of RA-FLSs on bone loss that might be important in RA pathogenesis.

Further, we analyzed the RA-FLS and BM-MSC datasets from GEO database trying to find the underlying mechanisms of RA-FLS on the balance of osteoblast and adipocyte differentiation capacity during low oxygen concentration. The bioinformatics analyses identified, LEP (gene encoding leptin) as the only overlapping upregulated gene among the DEGs from three independent comparisons.

The leptin protein is a hormone secreted by mature adipocytes [23]. Previous studies reported the inconsistent influences of leptin in bone homeostasis. A study by Ducy et al. using leptin*-*deficient mice demonstrated that the *in vivo* effects of leptin on hypothalamic-osteoblast loop contributed to bone loss [24]. Later, Hamrick et al. used the same animal model and found that leptin increased osteogenesis and limited adipogenesis [25]. Moreover, some studies demonstrated an increase of serum leptin levels in RA patients, and a positive correlation of leptin expression level with RA disease severity [26–28]. Increased hypoxia-mediated leptin expression in RA-FLSs was reported [29], Larsen et al. reported that the increase of leptin in RA-FLSs mainly depended on HIF-1 in hypoxia [30]. Our study showed a significant increase of leptin expression in RA-FLSs during hypoxia compared to that in OA-FLSs. In addition, we found leptin upregulation in RA-FLSs following osteogenic or adipogenic differentiation under hypoxia conditions. Thus, leptin might play a crucial role in the regulation of the balance of RA-FLS osteogenesis and adipogenesis during hypoxia.

A previous study demonstrated that the secreted leptin protein bound to the leptin receptor of BM-MSCs and enhanced adipogenesis but attenuated osteogenesis [31]. In addition, leptin was found to promote the IL-6 production via activation of JAK2-STAT3 pathway in RA-FLSs [32]. IL-6 mediated the differentiation in the early stages but initiated apoptosis in the later stage of osteogenesis [33], and inhibition of JAK reduced IL-6 production and increased bone formation in RA [34]. It suggested that leptin might regulate the imbalanced differentiation by JAK2-STAT3 pathway activation and IL-6 secretion. However, the inhibition of JAK had many side effects that limit the clinical application [35]. In this study, we found that leptin knockdown in RA-FLSs could reverse the hypoxia-mediated reduction of osteogenic differentiation, but attenuated the increase of adipogenic differentiation, which demonstrated the direct effect of leptin in the regulation of the balance of RA-FLS osteogenesis and adipogenesis during hypoxia. Therefore, leptin knockdown reversal of hypoxia-mediated reduction of RA-FLS osteogenic differentiation and increase of adipogenic differentiation could be a future effective therapeutic approach for repair of bone loss and reduction ofthe adipose formation in RA patients.

## 5. Conclusion

This study found that hypoxia regulation of leptin expression in RA-FLSs reduced the osteogenesis but increased the adipogenesis, which might be associated with RA pathogenesis. Therefore, target leptin might offer a novel strategy to improve RA therapy.

## Figures and Tables

**Table 1:** The primer sequences for RT-qPCR.

Gene	Primer sequences	
Leptin	Forward	GCTGTGCCCATCCAAAAAGTCC
	Reverse	CCCAGGAATGAAGTCCAAACCG

RUNX2	Forward	CCCAGTATGAGAGTAGGTGTCC
	Reverse	GGGTAAGACTGGTCATAGGACC

ALP	Forward	AGCGTGACTTGAAGTGTTGCATG
	Reverse	GAAAGGACCTGGACCACACAGA

OCN	Forward	CGCTACCTGTATCAATGGCTGG
	Reverse	CTCCTGAAAGCCGATGTGGTCA

FABP4	Forward	ACGAGAGGATGATAAACTGGTGG
	Reverse	GCGAACTTCAGTCCAGGTCAAC

C/EBP*α*	Forward	AGGAGGATGAAGCCAAGCAGCT
	Reverse	AGTGCGCGATCTGGAACTGCAG

PPAR*γ*	Forward	AGCCTGCGAAAGCCTTTTGGTG
	Reverse	GGCTTCACATTCAGCAAACCTGG

*β*-Actin	Forward	CACCATTGGCAATGAGCGGTTC
	Reverse	AGGTCTTTGCGGATGTCCACGT

## Data Availability

The supporting data in this study are available upon request.

## Supplementary Materials

Supplementary Materials Figure S1: the flowchart of the bioinformatics analysis procedure. The DEGs from GSE21959, GSE32006, and GSE55875 were selected with the criteria of an adjusted *p* value of <0.05 and |log2 (fold change)| ≥ 2 on the Sangerbox software. Then, the overlapping genes were showed with Venn, a web-based online tool.
