# Supplementary figures and images for: Hypoxia Inhibits Osteogenesis and Promotes Adipogenesis of Fibroblast-like Synoviocytes via Upregulation of Leptin in Patients with Rheumatoid Arthritis
Source: J Immunol Res. 2022 Dec 7;2022:1431399. doi: 10.1155/2022/1431399 (PMC9750767; doi:10.1155/2022/1431399)

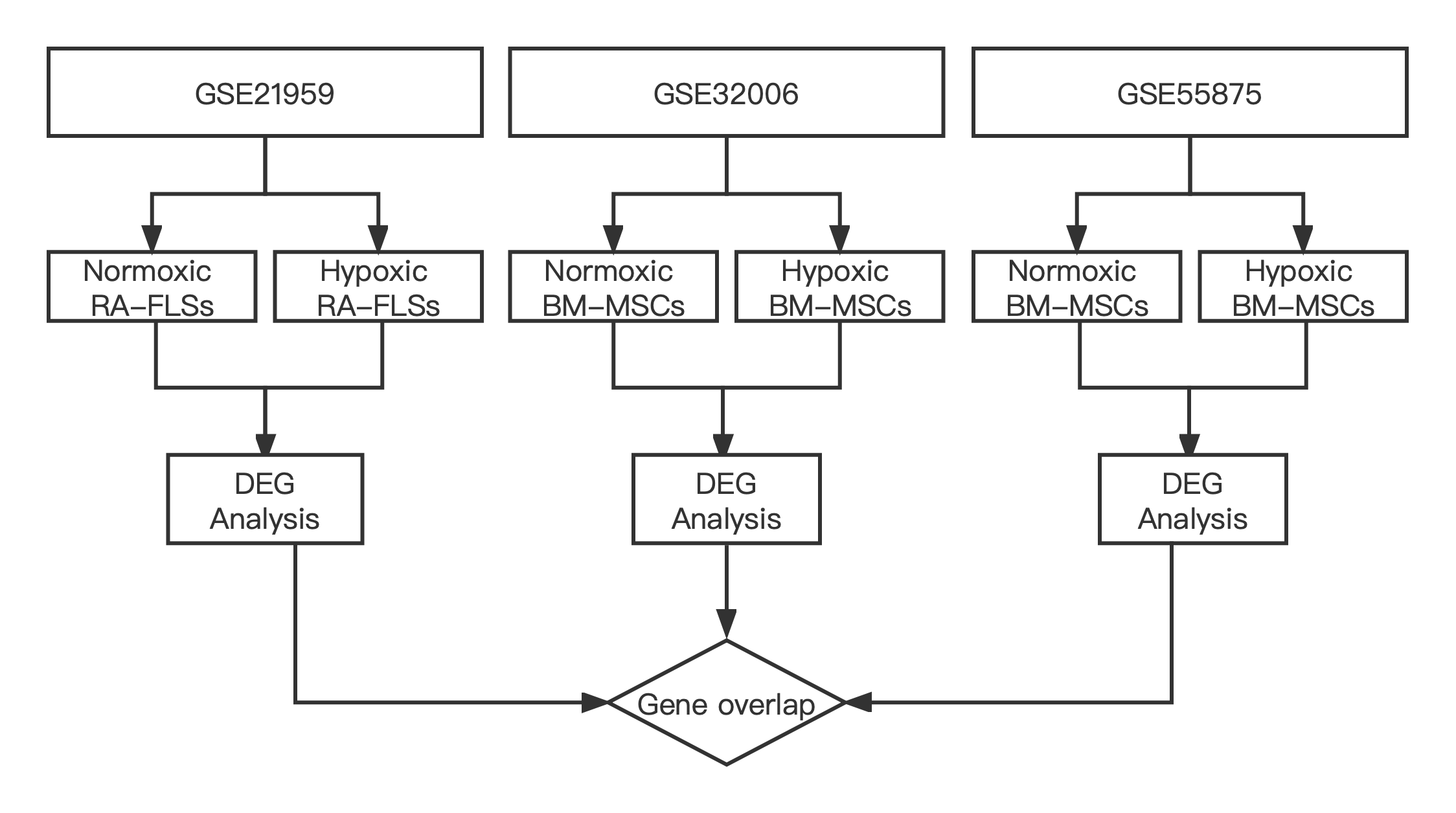

Supplement: Supplementary Materials — Figure S1: the flowchart of the bioinformatics analysis procedure. The DEGs from GSE21959, GSE32006, and GSE55875 were selected with the criteria of an adjusted p value of <0.05 and |log2 (fold change)| ≥ 2 on the Sangerbox software. Then, the overlapping genes were showed with Venn, a web-based online tool. [file 1431399.f1.docx]
